# Ultrasound single-phase CBE imaging for monitoring radiofrequency ablation of the liver tumor: A preliminary clinical validation

**DOI:** 10.3389/fonc.2022.894246

**Published:** 2022-07-22

**Authors:** Chiao-Yin Wang, Zhuhuang Zhou, Yu-Hsuan Chang, Ming-Chih Ho, Chiu-Min Lu, Chih-Horng Wu, Po-Hsiang Tsui

**Affiliations:** ^1^ Department of Medical Imaging and Radiological Sciences, College of Medicine, Chang Gung University, Taoyuan, Taiwan; ^2^ Department of Biomedical Engineering, Faculty of Environment and Life, Beijing University of Technology, Beijing, China; ^3^ Department of Medical Imaging, National Taiwan University Hospital, Taipei, Taiwan; ^4^ Departments of Surgery, National Taiwan University Hospital and College of Medicine, National Taiwan University, Taipei, Taiwan; ^5^ Center for Functional Image and Interventional Image, National Taiwan University, Taipei, Taiwan; ^6^ Department of Surgery, National Taiwan University Hospital Hsin-Chu Biomedical Park Branch, Hsin-Chu, Taiwan; ^7^ Department of Radiology, College of Medicine, National Taiwan University, Taipei, Taiwan; ^8^ Division of Pediatric Gastroenterology, Department of Pediatrics, Chang Gung Memorial Hospital at Linkou, Taoyuan, Taiwan; ^9^ Department of Biomedical Engineering, Chang Gung University, Taoyuan, Taiwan

**Keywords:** ultrasound, radiofrequency ablation, CBE imaging, liver tumor, HCC (hepatic cellular carcinoma)

## Abstract

Radiofrequency ablation (RFA) is an alternative treatment for early-stage hepatocellular carcinoma (HCC). The production of gas bubbles by RFA indicates threshold temperature of tissue necrosis and results in changes in backscattered energy (CBE) when ultrasound monitors RFA. In this study, ultrasound single-phase CBE imaging was used as a means of monitoring RFA of the liver tumor by analyzing the backscattering of ultrasound from gas bubbles in the liver. A total of 19 HCC patients were enrolled in the study. An ultrasound system was used during RFA to monitor the ablation process and acquire raw image data consisting of backscattered signals for single-phase CBE imaging. On the basis of single-phase CBE imaging, the area corresponding to the range of gas bubbles was compared with the tumor sizes and ablation zones estimated from computed tomography. During RFA, ultrasound single-phase CBE imaging enabled improved visualization of gas bubbles. Measured gas bubble areas by CBE were related to tumor size (the Spearman correlation coefficient *r*_s_ = 0.86; *p* < 0.05); less dependent on the ablation zone. Approximately 95% of the data fell within the limits of agreement in Bland-Altman plots, and 58% of the data fell within the 95% CI. This study suggests that single-phase CBE imaging provides information about liver tumor size because of the abundant vessels in liver tumors that promote the generation of gas bubbles, which serve as natural contrast agents in RFAs to enhance ultrasound backscattering. Ultrasound single-phase CBE imaging may allow clinicians to determine if the required minimum RFA efficacy level is reached by assessing gas bubbles in the liver tumors.

## Introduction

The most common form of liver cancer is hepatocellular carcinoma (HCC) (1, 2). Surgical resection and liver transplantation are the two main treatment options for HCC, depending on whether the patient is a suitable transplant candidate (3). HCC patients who are ineligible for surgery or liver transplantation may choose to undergo radiofrequency ablation (RFA) or microwave ablation (MWA), alternative therapies with a minimal invasiveness (3). RFA and MWA have similar therapeutic effects, complication rates, and rates of residual foci of untreated disease; however, RFA can be applied to tumor ablation with fewer sessions (4) and was recommended for safe and effective first-line treatment of early-stage HCC (5–7).

Physicians typically use computed tomography (CT) or ultrasound imaging guidance to place a needle electrode into a liver tumor (8, 9). A contrast-enhanced CT could further be used to monitor the progress of RFA and to assess its efficacy (10). Compared to CT, ultrasound provides a more portable method of guiding needle electrode insertion without radiation concerns. Notably, ultrasound can be difficult to detect the ablation zone for the following reasons: RFA heats up the tissue nearly to boiling point, resulting in gas bubbles which degrade image quality and obscure the ablation zone (11, 12). This is because gas bubbles are acoustically strong scatterers that contribute significant backscattered echoes when interacting with ultrasound. However, studies have shown that the areas of gas bubbles under high-temperature RFA correlates with those being treated by RFA (13–16), implying that the quantitative information obtained from temperature distribution and gas bubbles may be helpful in achieving ultrasound-guided RFA with intraoperative feedback of ablation zone measurements. Therefore, ultrasound monitoring of gas bubbles is critically meaningful and of potential during RFA in spite of not being widely used in clinical applications yet.

Echo time shift, changes in the backscattered energy (CBE), statistics of backscattered signals, and nonlinear parameters of the medium are commonly seen acoustic parameters that assist in the estimation of ultrasound temperature (17). Due to its recent improvements in technical developments and characteristics as described below, CBE may have greater potential in monitoring RFA of liver tumors. The CBE method was initially developed for noninvasive thermometry (18). The underlying mechanism for the temperature dependence of CBE is explained by thermal effects on the scatterers’ backscatter coefficients (19, 20). The accurate estimation and imaging of CBE require corrections for temperature-related signal motion effects (i.e., echo time shift); however, motion compensation is not necessary if CBE imaging is positioned solely to visualize thermal distribution (21). In order to use uncompensated CBE to monitor nonuniform heating with an improved contrast resolution and lower computational complexity, integrated CBE imaging (ICBE) utilizing sliding window processing and a polynomial approximation has also been proposed (22). As CBE artifacts are prevalent at the location of the RFA electrode, a recent study proposed ultrasound single-phase CBE imaging based on positive CBE values; an *in vitro* validation demonstrated that single-phase CBE imaging suppressed artifacts and was more accurate in estimating the ablation zone (23). Moreover, RFA-induced gas bubbles may be used as natural contrast agents in CBE imaging, as changes in the level of backscattered signals are susceptible to tissue-air interfaces due to their large difference in acoustic impedance. Therefore, ultrasound single-phase CBE imaging could be a feasible strategy for intraoperative monitoring of clinical RFA for liver tumors.

This study aims to evaluate the effectiveness of ultrasound single-phase CBE imaging in monitoring RFA treatment of liver tumors for HCC patients. To clarify the clinical relevance and position of ultrasound CBE, the range of gas bubbles measured by the proposed method is compared with the tumor size and the ablation zone.

## Materials and methods

### Subjects

The Institute Review Board (IRB) of National Taiwan University Hospital (NTUH) approved this study (IRB number: 201804053RINC). Subjects signed informed consents and experiments were conducted in accordance with approved protocols. In total, 19 patients (age: 62.3 ± 11.5 years, range: 42 to 88 years) with newly diagnosed HCC with the Milan criteria who are scheduled for RFA treatments have been recruited, and their demographic information is shown in **Table 1**.

**Table 1:** Demographic data of the patients and RFA parameters used in the study.

Characteristics	
**No. of participants**	19
**Age, years**	
Mean ± standard deviation (range)	62.1 ± 12.1 (42.0 – 88.0)
Median	64.0
**Tumor size (mm^2^)**	
Mean ± standard deviation (range)	159.4 ± 78.4 (71.1 – 427.4)
Median	144.0
**Heating conditions**	
**Power range, watt**	
Min – max	56 – 140
**Last tip temperature, °C**	
Mean ± standard deviation (range)	79.8 ± 9.1 (52 – 92)
Median	82.0
**Heating time, minute**	
Mean ± standard deviation (range)	10.2 ± 1.7 (6 – 12)
Median	11

### Experimental procedures

**Figure 1:**
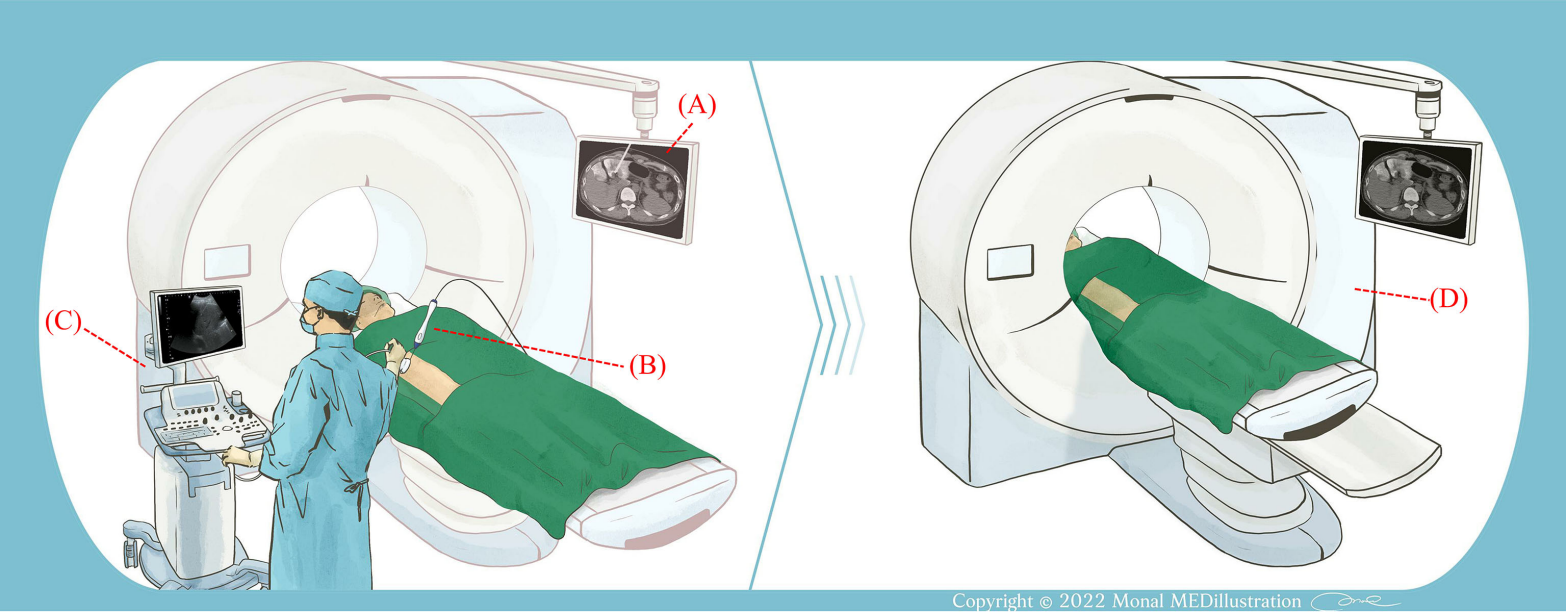
Illustration of the experimental procedure. **(A)** CT was used to measure the size of the liver tumor and guide the placement of the RF needle electrode. **(B)** After determining the electrode position, the RFA system was activated and ablation was performed. **(C)** An ultrasound system was used to monitor RFA. The ultrasound transducer was held in a freehand fashion. **(D)** Following RFA, the patient received a contrast injection and CT scans were performed to examine the ablation area.

Refer to **Figure 1** for experimental procedures. For each patient, CT-guided RFA using iodized oil (Lipiodol, Andre Guerbet, Aulnay-sous-Bois, France) was performed as described in the previous study (24). The tumor size was measured firstly. Depending on the size of the tumor, approximately 2 to 5 mL of iodized oil was applied. An abdominal angiography was conducted, and the infused iodized oil was observed through fluoroscopy until the tumor staining was determined. Afterwards, the patient was transferred to a CT room (Ingenuity 128 CT, Philips, Amsterdam, Netherlands), placed supine, and underwent anesthesia. A commercially available RFA system (Cool-tip, Covidien, Mansfield, MA, USA; BigTip & V-tip, RF Medical, Seoul, Korea; VIVA RF generator, STARmed, Goyang, Korea; LeVeen, Boston Scientific, Natick, MA, USA) was used in the study. Several CT scans were performed to confirm the position of the RFA needle using a 21-gauge Chiba needle. Using a needle electrode (2 cm or 3 cm active tip), tumor ablations were performed until the ablation zones cover the entire tumor.

**Figure 2:**
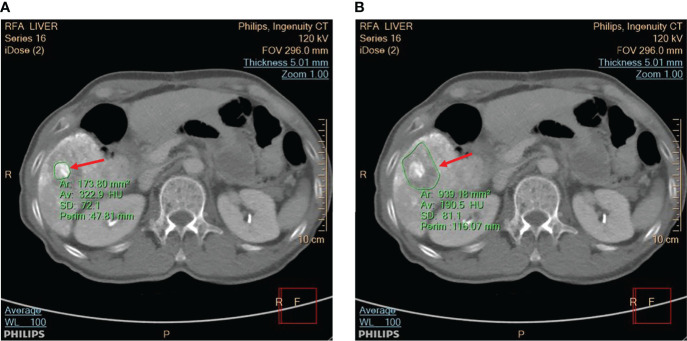
The CT scan was utilized to view **(A)** the tumor and **(B)** the ablation zone, as indicated by red arrows. The application IntelliSpace Portal (Philips) was utilized to measure the tumor size and ablation zone size, as shown in the CT images segmented by green contour lines.

During the RFA procedure, an ultrasound scanner (Model 2000, Terason, Burlington, MA, USA) and convex array transducer (Model 10L5, Terason) were used to monitor the ablation by an experienced radiologist. The ultrasound transducer was placed axially along the RF electrode and held in a freehand fashion without movement in order to ensure that the needle tip would be clearly visible in the B-scan. The raw data consisted of 256 scan lines of ultrasound backscattered signals at the sampling rate of 12 MHz which were acquired every minute until the RFA was completed. After this, the patient was scanned with a contrast-enhanced CT and the application IntelliSpace Portal (Philips), which is part of the workstation, was used to determine the ablation zone, as shown in **Figure 2**.

It should be noted that the inflammatory response will cause peripheral rim enhancement in hepatic artery phase CT images. Therefore, to minimize the effect of inflammation, porto-venous phase images were used to measure tumor sizes and ablation zones. Moreover, CT and contrast-enhanced ultrasound (CEUS) images can be applied to post-RFA evaluations. However, the CEUS images have relatively lower spatial resolution; comparatively, CT images are still regarded as the gold standard for measuring tumors and ablation zones.

### Ultrasound single-phase CBE imaging

**Figure 3:**
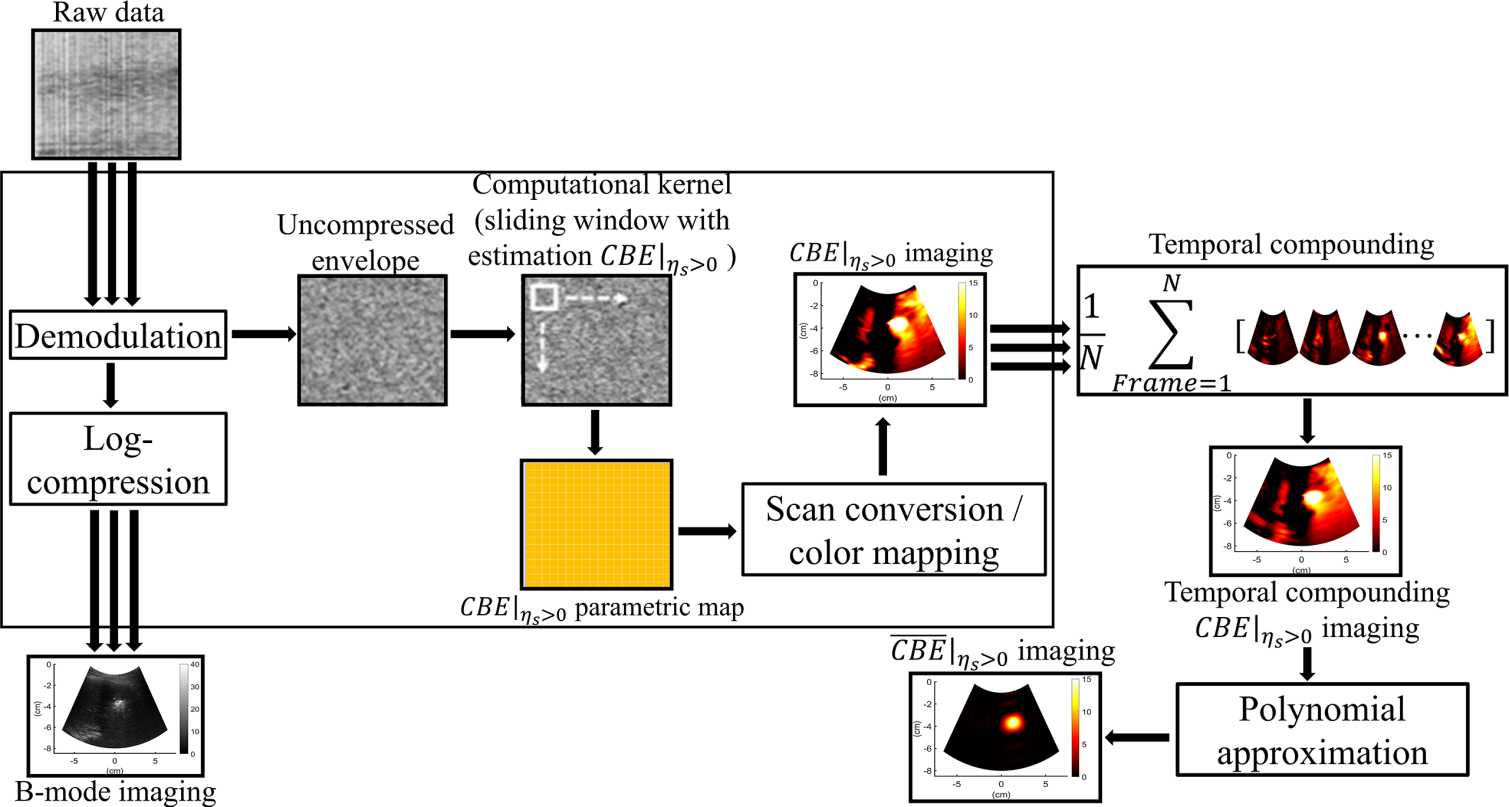
The algorithmic scheme for ultrasound single-phase CBE imaging. The uncompressed envelope signals were processed by the sliding window technique to obtain *CBE*|_*η*_*s*_>0_ images at different time points, which were further combined by temporal compounding. Polynomial approximation of the temporal compounding *CBE*|_*η*_*s*_>0_ image (denoted by ${\bar{CBE}|}_{\eta_{s}>0}$ ) was subsequently applied to visualize temperature distributions and heat conduction behaviors.

Raw data from the image was then used for ultrasound single-phase CBE imaging. In contrast to conventional CBE imaging which derives its information from the ratio of backscattered energy at each temperature relative to the reference at each pixel (20, 21), single-phase CBE imaging is based on a window-to-window computational scheme which reduces the effects of speckle motion and simplifies the algorithm for practical applications (23). It is illustrated in **Figure 3** and briefly described below how the detailed algorithm described in the previous study (23) operates.

Each raw data set was processed to form an envelope image using an analytic expression for the backscattered signal, and the corresponding B-mode image was constructed using logarithm-compressed envelope images with a dynamic range of 40 dB. Initially, a window is positioned at the upper-left corner of the uncompressed envelope image *R_k_* at each time point (*k* = 0, 1, 2, … min; *R*_0_ is the preablation data) for acquiring local envelope data $\overset{⌢}{R_{k}}$ . The regional CBE value (denoted by *η_s_*) is calculated using equation (1) and assigned as the new pixel corresponding to the window location.


(1) $$\eta_{s}=10\cdot log_{10}\left(\frac{E\;\left[{\hat{R}}_{k}^{2}\right]}{E\;\left[{\hat{R}}_{0}^{2}\right]}\right).$$

where *E*[·] denote the statistical mean. Let the window move throughout *R*_k_ and *R*_0_ in steps of a certain number of pixels corresponding to a window overlap ratio (WOR) for calculating regional CBE values. After data interpolation, a CBE image with the same size as the original image can be obtained. It should be noted that both positive and negative pixels (denoted by *CBE*|_*η*_*s*_>0_ and *CBE*|_*η*_*s*_<0_, respectively) simultaneously exist in a CBE image. Single-phase CBE imaging was defined as *CBE*|_*η*_*s*_>0_ parametric imaging, which was achieved by adjusting negative values in CBE image 0. Using a technique known as temporal compounding, we were able to collect sufficient information about backscattering from gas bubbles and improve the visualization of ablation zones (25); that is, *CBE*|_*η*_*s*_>0_ maps acquired at various time points are used for summing and averaging to obtain the temporal compounding *CBE*|_*η*_*s*_>0_ imaging. Polynomial approximation of the temporal compounding *CBE*|_*η*_*s*_>0_ image (denoted by ${\bar{CBE}|}_{\eta_{s}>0}$ ) was subsequently applied to visualize temperature distributions and heat conduction behaviors.

In the computational settings, the order of performing polynomial approximation was set to 12, based on empirical data previously obtained (23). The WOR was set to 50%, and the side of the sliding window was three times the pulse length of the ultrasound transducer (6.9 mm).

### Data analysis and statistical analysis

In the analysis of ${\bar{CBE}|}_{\eta_{s}>0}$ images, the areas within the contours of −1 to −6 dB were segmented to qualitatively measure the regions of shading change within the single-phase CBE image (denoted by $S_{{\bar{CBE}|}_{\eta_{s}>0}}$ with mm^2^ as the unit). The values of $S_{{\bar{CBE}|}_{\eta_{s}>0}}$ were compared with those of tumor sizes and ablation zone sizes using Spearman correlation coefficients *r*_s_ (significant differences were identified at *p* < 0.05). Furthermore, data of $S_{{\bar{CBE}|}_{\eta_{s}>0}}$ , the tumor sizes, and the ablation zone sizes were compared by using paired sample *t* test, and the Bland-Altman plot was used to compare measurements $S_{{\bar{CBE}|}_{\eta_{s}>0}}$ with tumor size and ablation zone size, respectively, in order to evaluate the applicability of ultrasound single-phase CBE imaging in monitoring RFA. Statistical analyses were conducted using SigmaPlot 14.0 (Systat Software, Inc., CA, USA) and MedCalc software (MedCalc Software Ltd, Ostend, Belgium).

## Results

**Figure 4:**
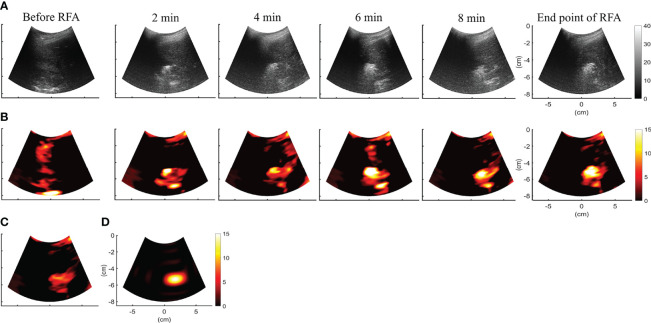
**(A, B)** depict the B-mode and *CBE*|_*η*_*s*_>0_ images obtained during RFA of the liver tumor at different time points. *CBE*|_*η*_*s*_>0_ images were further temporally combined as **(C)** *CBE*|_*η*_*s*_>0_ and **(D)** ${\bar{CBE}|}_{\eta_{s}>0}$ images, respectively. Compared with B-scan, ${\bar{CBE}|}_{\eta_{s}>0}$ imaging largely suppressed information related to nonablated tissues, and artifacts were also not found to enable estimations of the ablation zone.

**Figures 4A, B** depict the B-mode and *CBE*|_*η*_*s*_>0_ images obtained during RFA of the liver tumor at different time points. Due to the generation of gas bubbles associated with the ablation zone, hyperechoic areas were observed in the B-mode image. Although there was no significant change in the image brightness of the ablation zone over time, the spatial distribution of speckle patterns in hyperechoic areas seemed to have increased, presumably due to heat conduction in the liver tumor reflected in increased gas bubble levels. This phenomenon can be revealed in each *CBE*|_*η*_*s*_>0_ image, which were further combined as the temporal compounding *CBE*|_*η*_*s*_>0_ and ${\bar{CBE}|}_{\eta_{s}>0}$ images, as shown in **Figures 4C, D**, respectively. Compared with B-scan, ${\bar{CBE}|}_{\eta_{s}>0}$ imaging largely suppressed information related to nonablated tissues, and artifacts were also not found to enable estimations of the ablation zone.

**Figure 5:**
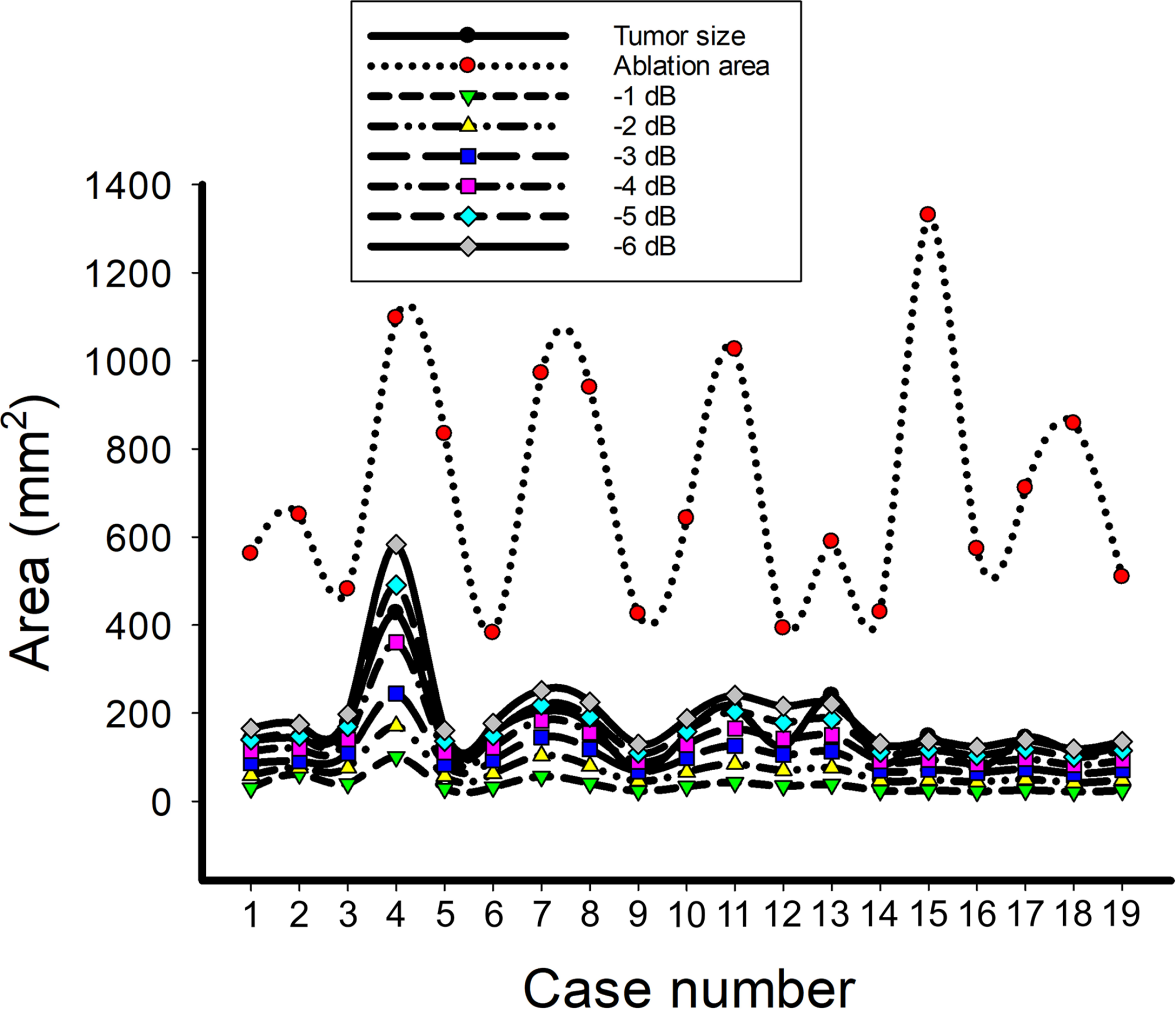
The data of the tumor size, ablation zone size, and $S_{{\bar{CBE}|}_{\eta_{s}>0}}$ for each subject estimated according to various contour criteria. By adjusting the criteria from −1 to −6 dB contours, $S_{{\bar{CBE}|}_{\eta_{s}>0}}$ increased accordingly and approximated the size of the tumor; nevertheless, the ablation zone size was smaller and less dependent on the ablation zone size.

**Figure 6:**
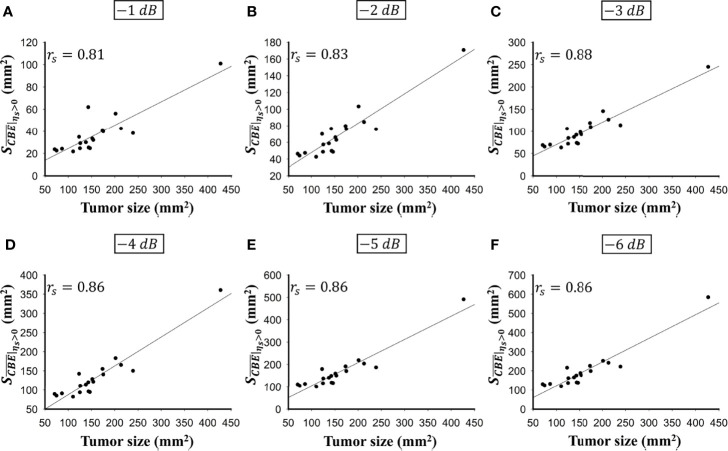
The relationship between the tumor size and $S_{{\bar{CBE}|}_{\eta_{s}>0}}$ obtained according to various contour criteria. **(A)** −1 dB; **(B)** −2 dB; **(C)** −3 dB; **(D)** −4 dB; **(E)** −5 dB; **(F)** −6 dB. The values of $S_{{\bar{CBE}|}_{\eta_{s}>0}}$ using various contour criteria correlated with the size of the liver tumor (*p* < 0.05; *r*_s_ = 0.81 to 0.86 corresponding to −1 to −6 dB contours).

**Figure 7:**
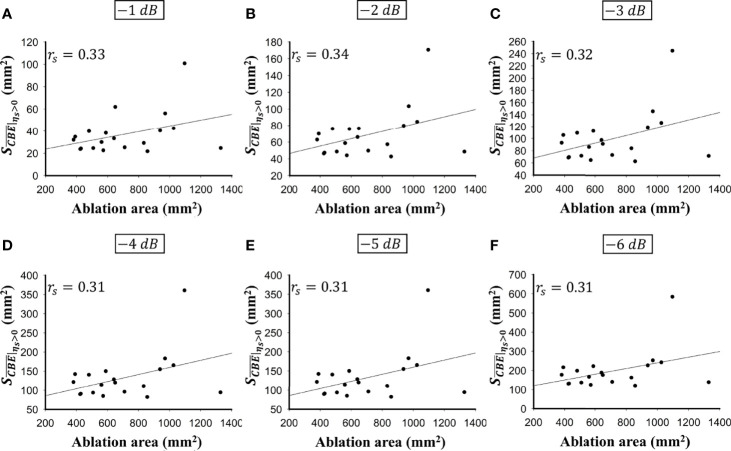
The relationship between the ablation zone size and $S_{{\bar{CBE}|}_{\eta_{s}>0}}$ obtained according to various contour criteria. **(A)** −1 dB; **(B)** −2 dB; **(C)** −3 dB; **(D)** −4 dB; **(E)** −5 dB; **(F)** −6 dB. The correlation *r*_s_ between $S_{{\bar{CBE}|}_{\eta_{s}>0}}$ and the ablation zone size were approximately 0.3 for each contour criterion, which indicates that $S_{{\bar{CBE}|}_{\eta_{s}>0}}$ is not able to characterize the ablation zone.

**Figure 8:**
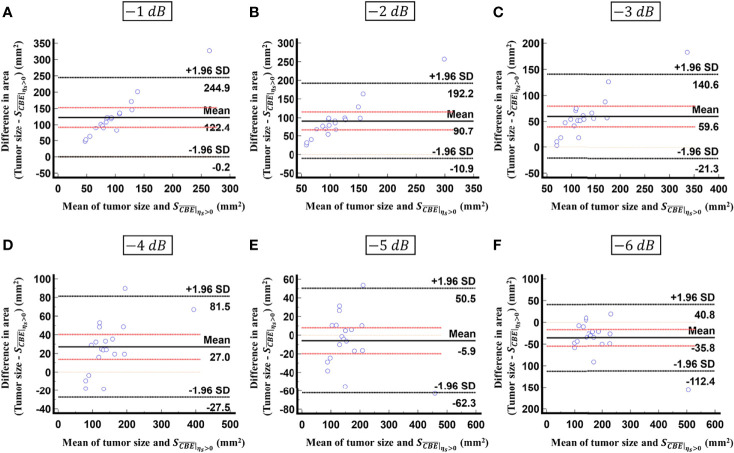
Bland-Altman plots of the differences between the tumor sizes and $S_{{\bar{CBE}|}_{\eta_{s}>0}}$ values against the averages of the two sets of measurements obtained according to various contour criteria. **(A)** −1 dB; **(B)** −2 dB; **(C)** −3 dB; **(D)** −4 dB; **(E)** −5 dB; **(F)** −6 dB. The red lines represent the 95% CI of the mean difference. The black lines mean the limits of agreement. About 95% of the data points fell within the limits of agreement; in particular, 58% of the data fell within the 95% CI when the contour criteria of −6 dB was used.

**Figure 9:**
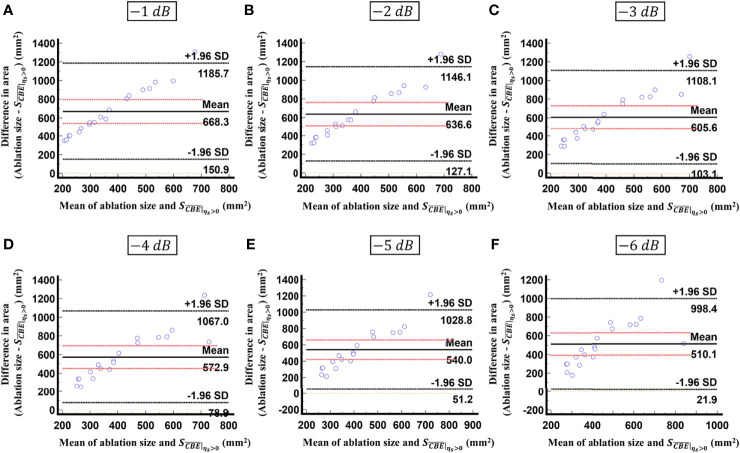
Bland-Altman plots of the differences between the ablation zone sizes and $S_{{\bar{CBE}|}_{\eta_{s}>0}}$ values against the averages of the two sets of measurements obtained according to various contour criteria. **(A)** −1 dB; **(B)** −2 dB; **(C)** −3 dB; **(D)** −4 dB; **(E)** −5 dB; **(F)** −6 dB. The red lines represent the 95% CI of the mean difference. The black lines mean the limits of agreement. In comparison with the results in **Figure 8**, less than 30% of the data fitted into the 95% CI, indicating that $S_{{\bar{CBE}|}_{\eta_{s}>0}}$ is inappropriate for estimating the ablation zone size.

**Figure 5** shows the tumor size, ablation zone size, and $S_{{\bar{CBE}|}_{\eta_{s}>0}}$ for each subject estimated according to various contour criteria. By adjusting the criteria from −1 to −6 dB contours, $S_{{\bar{CBE}|}_{\eta_{s}>0}}$ increased accordingly and approximated the size of the tumor. However, both tumor sizes and $S_{{\bar{CBE}|}_{\eta_{s}>0}}$ were smaller than the ablation zone sizes, suggesting that the liver tumors were completely covered by the ablation zones; $S_{{\bar{CBE}|}_{\eta_{s}>0}}$ was less dependent on the size of the ablation zone. **Figures 6**, **7** illustrate the dependence of $S_{{\bar{CBE}|}_{\eta_{s}>0}}$ , respectively, on the size of the ablation zone and the size of the tumor. The values of $S_{{\bar{CBE}|}_{\eta_{s}>0}}$ using various contour criteria correlated with the size of the liver tumor (*p* < 0.05; *r*_s_ = 0.81 to 0.86 corresponding to −1 to −6 dB contours). No significant differences between $S_{{\bar{CBE}|}_{\eta_{s}>0}}$ (−4 to −6 dB contours) and the tumor size were found, as shown in **Table 2**. Comparatively, *r*_s_ between $S_{{\bar{CBE}|}_{\eta_{s}>0}}$ and the ablation zone size were approximately 0.3 for each contour criterion, which indicates that $S_{{\bar{CBE}|}_{\eta_{s}>0}}$ is not able to characterize the ablation zone. **Figure 8** shows the Bland-Altman plots of the differences between the tumor sizes and $S_{{\bar{CBE}|}_{\eta_{s}>0}}$ values against the averages of the two sets of measurements. The red lines represent the 95% confidence interval (CI) of the mean difference. The black lines mean the limits of agreement, which are defined as the mean difference plus and minus 1.96 times the standard deviation of the differences. About 95% of the data points fell within the limits of agreement, indicating good agreement between real tumor size and the value estimated by ultrasound single-phase CBE imaging. In particular, 58% of the data fell within the 95% CI when the contour criteria of −6 dB was used. In contrast, less than 30% of the data fitted into the 95% CI when Bland-Altman plots were used to compare the ablation zone size and $S_{{\bar{CBE}|}_{\eta_{s}>0}}$ , as shown in **Figure 9**. According to the correlation analysis and Bland-Altman plots, the contour criteria of −6 dB was suggested for using single-phase CBE imaging to measure tumor size.

**Table 2:** Comparisons of data between $S_{{\bar{CBE}|}_{\eta_{s}>0}}$ (−1 to −6 dB contours), the tumor sizes, and the ablation zone sizes by using paired sample *t* test.

	Contour of $S_{{\bar{CBE}|}_{\eta_{s}>0}}$					
	-1 dB	-2 dB	-3 dB	-4 dB	-5 dB	-6 dB
Tumor size	9×10^-7*^	4×10^-5*^	3×10^-3*^	0.12	0.41	0.12
Ablation area	1×10^-9*^	2×10^-9*^	5×10^-9*^	9×10^-9*^	1×10^-8*^	4×10^-8*^

^*^No significant differences between $S_{{\bar{CBE}|}_{\eta_{s}>0}}$ (−4 to −6 dB contours) and the tumor size were found, representing that ultrasound single-phase CBE imaging reliably measured tumor size.p < 0.05 significant difference.

## Discussion

A successful RFA relies on the application of a thermal dose sufficient to cause coagulation necrosis of the liver tumor (26). Local tumor progression rates may be reduced by generating an adequate ablation zone surrounding the target tumor (27). Therefore, intraoperative monitoring of RFA and estimating the size of the ablation zone and the range of the target tumor should be considered as pointers that clinicians can use to make more precise evaluations of RFA effectiveness. In light of the advances made in CBE imaging, it is now possible to monitor RFA by ultrasound in a clinical setting. During this study, we validated the use of ultrasound single-phase CBE imaging for monitoring RFA of liver tumors. It has been demonstrated that ultrasound single-phase CBE imaging offers better visualization of gas bubbles generated during RFA than conventional B-scan. Further, the assessment of the spatial distribution of gas bubbles according to single-phase ultrasound CBE imaging directly correlated with tumor size; less dependence was seen on the ablation zone. This is the first study that reports *in vivo* CBE-based imaging for the clinical assessment of hepatic tumors. The following sections will discuss physical interpretations, possible underlying mechanisms, implications, applications, and limitations.

According to two kinds of existing theories, the physical meanings of ultrasound single-phase CBE imaging in the RFA procedure are involved in a number of effects. Straube and Arthur (18) proposed their first theory whereby the behavior of CBE is determined by the properties of scatterers (i.e., the thermal effects on the backscatter coefficient). When the temperature increases, the backscattering energies measured from lipid-based scatterers linearly increase, and those returned from aqueous-based scatterers linearly decrease (18–20). Besides temperature, CBE sensitivity is also affected by ultrasound frequency (28), which is an important factor affecting ultrasound backscattering strength (29). CBE is partially explained by the second theory (30), where local changes in speckle patterns are caused by thermal effects to further alter the sound speed and the waveform features of the backscattered ultrasound signal. However, we should note that the above interpretation models are only applicable for temperatures between 30°C and 50°C. Currently, there is no appropriate model to explain the behavior of CBE imaging at high temperatures; however, the previous study suggested that stiffness increases, tissue necrosis, and gas bubble formation might be dominant reasons for CBE under high-temperature RFA (23).

Studies have indicated that the spatial distribution of gas bubbles caused by RFA correlates with the size of the ablation zone (13–16). An *in vitro* study using porcine muscle samples has shown that the range of gas bubbles corresponds to the ablation zone (31). Validation *in vitro* using the porcine liver model also demonstrated that the area of gas bubble distribution observed on ultrasound single-phase CBE imaging was correlated with the size of the ablation zone (23). However, clinical validation in this study indicated that ultrasound single-phase CBE imaging reflected tumor size rather than ablation zone size. Discrepancy between this finding and previous reports should be discussed for clarification of considerations regarding the proposed method in clinical applications. For the liver, when the tissue temperature rises above 60°C and remains for a few seconds, irreversible damage may occur due to coagulation necrosis (32). A temperature of 60°C was also a critical temperature for generating gas bubbles (31). Therefore, gas bubbles may be considered as a sign of coagulation necrosis.

Note that water content is related to the efficiency of gas bubble formation to some extent, since gas bubbles are a direct result of water vaporization under high-temperature ablation. As previously noted, liver cancer growth requires the formation of new blood vessels (angiogenesis) (33) and HCC is a typically hyper-vascular tumor that exhibits abundant and tortuous vessels (34). Consequently, liver tumors have a relatively high water content, which facilitates significant gas bubble formation when the temperature reaches the threshold level. In practice, a complete ablation zone includes the target tumor that is heated, as well as an adequate margin that is free of tumor tissue for the successful completion of RFA (35). In comparison with the liver tumor, the density of the vascular structures in non-tumor tissues may be relatively low, resulting in less concentration of gas bubbles during RFA, which cannot contribute significant backscattered signals for CBE imaging. Single-phase CBE is unable to describe the ablation zone accurately, however, its ability to depict the tumor size may be considered as a new strategy to evaluate RFA. As the tumor size estimated by ultrasound single-phase CBE imaging corresponds to CT examinations prior to RFA, it indicates that the thermal dose distributed within the target tumor is sufficient to generate gas bubbles, which represent tissue necrosis and fulfill the minimum RFA efficacy requirement.

The study has some limitations. The first issue is that the number of patients is not sufficient. There is a need to conduct large-scale clinical trials to more precisely calibrate the correlation between tumor sizes obtained from ultrasound single-phase CBE imaging and CT scans. Additionally, the proposed CBE technique is based on the analysis of ultrasound backscattered echoes returned from gas bubbles, which are however not available in residual tumors due to insufficient thermal dose or unsuccessful ablation. Under this circumstance, CBE is unable to detect residual tumors. Also, CBE-based imaging may not be suitable for monitoring RFA of lower water-content tissues that do not generate gas bubbles easily. Third, freehand handling of the transducer may result in measurement error. Further development of a probe fixer or RF needle guide attached to the ultrasound transducer is needed to improve measurement accuracy by increasing needle visibility and stability. In addition, the monitoring of RFA using single-phase CBE requires an ultrasound imaging system capable of accessing raw image data. The majority of clinical systems are unable to output raw data, so further development of algorithms for ultrasound CBE imaging using B-scan data may be needed to facilitate clinical applications.

In conclusion, the single-phase CBE method is able to detect gas bubbles, which serve as natural contrast agents during RFA to enhance ultrasound backscattering, enabling the use of ultrasound imaging to estimate the tumor size and establish whether the minimum level of RFA efficiency has been achieved.

## Data availability statement

The original contributions presented in the study are included in the article/supplementary material, Further inquiries can be directed to the corresponding authors.

## Ethics statement

The studies involving human participants were reviewed and approved by the Institute Review Board (IRB) of National Taiwan University Hospital (NTUH) approved this study (IRB number: 201804053RINC). The patients/participants provided their written informed consent to participate in this study.

## Author contributions

C-YW and ZZ are equally contributed. C-HW and P-HT contributed to this paper with conception and design. C-YW collected the data and performed ultrasound scan. Y-HC and M-CH performed CT scan and provided interpretation. C-YW, ZZ, and C-ML performed image and statistical analysis. C-YW, ZZ, and P-HT drafted the manuscript. C-HW contributed revision. All authors contributed to the article and approved the submitted version.

## Funding

This work was supported by the Ministry of Science and Technology in Taiwan (Grant No. MOST 109-2223-E-182-001-MY3), Chang Gung Memorial Hospital at Linkou in Taiwan (Grant Nos. CMRPD1L0251 and CMRPD1L0081) and National Taiwan University Hospital (Grant No. 108-M4430 and 109-M4557).

## Conflict of interest

The authors declare that the research was conducted in the absence of any commercial or financial relationships that could be construed as a potential conflict of interest.

## Publisher’s note

All claims expressed in this article are solely those of the authors and do not necessarily represent those of their affiliated organizations, or those of the publisher, the editors and the reviewers. Any product that may be evaluated in this article, or claim that may be made by its manufacturer, is not guaranteed or endorsed by the publisher.
